# Molecular anatomy of the subcellular localization and nuclear import mechanism of herpes simplex virus 1 UL6

**DOI:** 10.18632/aging.102965

**Published:** 2020-04-01

**Authors:** Mingsheng Cai, Xiaowen Ou, Yiwen Li, Xingmei Zou, Zuo Xu, Yuanfang Wang, Hao Peng, Yangxi Deng, Yingjie Guo, Manjiao Lu, Weidong Gan, Tao Peng, Meili Li

**Affiliations:** 1 Guangdong Provincial Key Laboratory of Allergy and Clinical Immunology, Second Affiliated Hospital of Guangzhou Medical University, Sino-French Hoffmann Institute, School of Basic Medical Science, Guangzhou Medical University, The Sixth Affiliated Hospital of Guangzhou Medical University, Qingyuan People’s Hospital, Guangzhou 510260, Guangdong, China; 2 State Key Laboratory of Respiratory Diseases, Sino-French Hoffmann Institute, School of Basic Medical Science, Guangzhou Medical University, Panyu, Guangzhou 511436, Guangdong, China; 3 South China Vaccine Corporation Limited, Guangzhou Science Park, Guangzhou 510663, Guangdong, China

**Keywords:** HSV-1, UL6, nuclear import, importin, Ran-GTP

## Abstract

As an indispensable structure protein, the herpes simplex virus 1 (HSV-1) UL6 has been described to exert numerous roles in viral proliferation. However, its exact subcellular localization and subcellular transport mechanism is not well known. In the present study, by utilizing confocal fluorescent microscopy, UL6 was shown to mainly locate in the nucleus in enhanced yellow fluorescent protein or Flag tag fused expression plasmid-transfected cells or HSV-1-infected cells, whereas its predicted nuclear localization signal was nonfunctional. In addition, by exploiting dominant negative mutant and inhibitor of different nuclear import receptors, as well as co-immunoprecipitation and RNA interference assays, UL6 was established to interact with importin α1, importin α7 and transportin-1 to mediate its nuclear translocation under the help of Ran-mediated GTP hydrolysis. Accordingly, these results will advance the knowledge of UL6-mediated biological significances in HSV-1 infection cycle.

## INTRODUCTION

Herpes simplex virus 1 (HSV-1), a large nuclear duplicating DNA virus, is an epidemic human microbe that can provoke a lytic infection in the mucosal epithelial cells but a life-long latent infection in neurons. As one of the fundamental structure proteins of HSV-1, UL6 has obtained remarkable concern by virtue of its association with numerous viral propagation processes, including establishing the portal for DNA entry into the HSV capsid, cleavage, processing and packaging of replicated viral DNA, assembling of a minor constituent of virions and capsids, and locating on the external surface of the viral capsid [1–6]. Besides, recent studies also showed that the tryptophan residues or putative leucine zipper of UL6 is crucial for its association with scaffold proteins, UL15 and UL28 proteins, as well as the incorporation of the portal into capsids [7–10]. However, the definite function of UL6 is still poorly understood.

As it is known to all, investigating the precise subcellular localization of a specific protein is a meaningful way to initially discern its detailed roles. UL6 has been previously demonstrated to target to the nuclei in chemical fixed cells [1, 4, 11, 12]. By employing the extensively used fluorescent microscopy technique [13–24], here we established that UL6 was principally localized to the nuclei in both transient transfected live and chemical fixed cells, as well as in HSV-1-infected cells. Furthermore, UL6 was demonstrated to be transported to the nucleus through a Ran-, importin α1-, importin α7- and transportin-1-dependent nuclear import mechanism, which was predominantly mediated by importin α7 and transportin-1.

## RESULTS AND DISCUSSION

### Subcellular localization of UL6 in the plasmid transfected and virus infected cells

**Figure 1:**
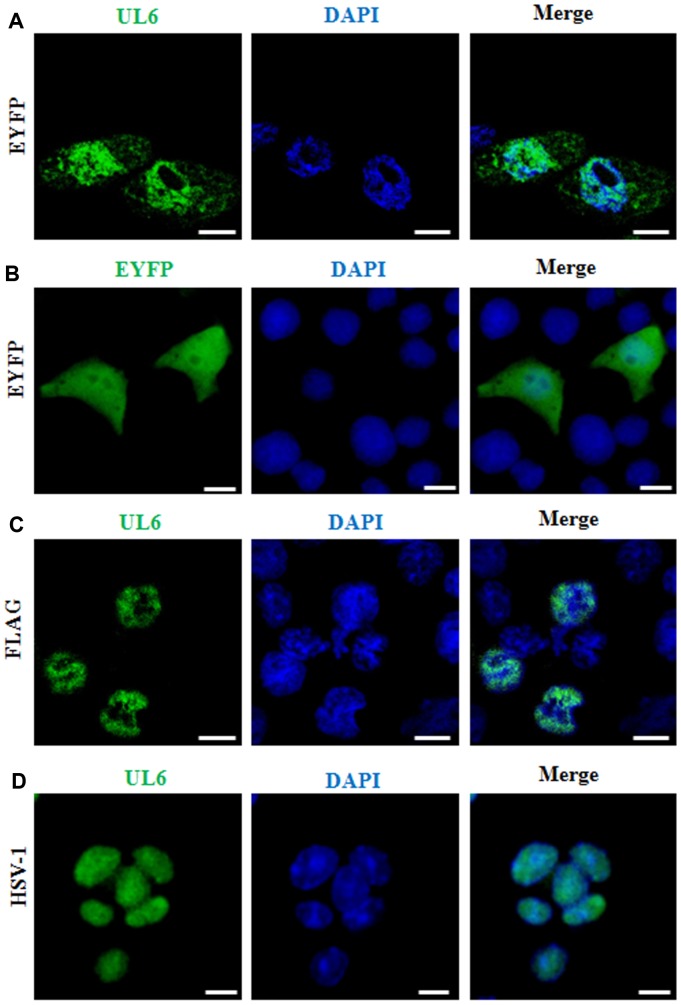
**Subcellular distribution of UL6 in plasmid-transfected and HSV-1-infected cells.** Subcellular distribution of EYFP-UL6 (**A**), EYFP (**B**) and FLAG-UL6 (**C**) in related plasmid transfected COS-7 cells. (**D**) Subcellular distribution of UL6 in HSV-1 infected Vero cells. Vero cells were infected with HSV-1 (F strain) at an MOI of 1. 8 h post-infection, Vero cells were fixed with 4% paraformaldehyde, permeabilized with 0.5% Triton X-100, and incubated with the anti-UL6 pAb. Then, cells were incubated with FITC-conjugated goat anti-rabbit IgG (green) and stained with DAPI (blue) to visualize the nuclei. EYFP fusion proteins were shown in pseudocolor green. The image shown represents a great proportion of the cells with homogeneous subcellular distribution. All scale bars indicate 10 um. Statistical analysis of the fluorescence was shown in Table 1.

Protein is the executor of life activity, which need to be transported into certain cell compartments for its execution of specific biological function. UL6 was previously demonstrated to localize in the nucleus in chemical fixed cells [1, 4, 11, 12]. To further detect the subcellular distribution of UL6 in plasmid transfected live cells, enhanced yellow fluorescent protein (EYFP)-tagged UL6 and confocal fluorescence microscopy were adapted. Subsequently, plasmid encoding UL6 fused to the C-terminus of EYFP was constructed and transfected into COS-7 cells to test the subcellular localization of UL6, without the presence of other HSV-1 constituents. Although EYFP-UL6 could show cytoplasmic or pan-cellular localization, it largely exhibited nuclear localization (Figure 1A and Table 1). On the contrary, the fluorescence of vector control EYFP was homogeneously dispersed throughout the cytoplasm and the nucleus in cells transfected with pEYFP-C1 (Figure 1B and Table 1).

**Table 1:** Subcellular localization of HSV-1 UL6.

**Transfection or infection**	**Detected protein**	**Total number of cells transfected with plasmid or infected with virus**	**Number of cells with predominant nuclear localization**	**Percentage of cells with predominant nuclear localization**
Transfected with EYFP-UL6	UL6	30	21	70
Transfected with EYFP vector	EYFP	30	0	0
Transfected with Flag-UL6	UL6	30	29	96.67
Infected with HSV-1	UL6	30	30	100

Since EYFP is a relatively considerable tag (~27 kDa), it may alter the nuclear localization of UL6. To avoid this hypothesis, plasmid encoding Flag-tagged UL6 (pCMV-Flag-UL6) was constructed and immunofluorescence assay (IFA) was performed to examine the subcellular localization of the UL6. As shown in Figure 1C and Table 1, Flag-tagged UL6 also localized in the nucleus following formaldehyde-based fixation method.

It is well known that viral protein may show distinct subcellular localization fashions in plasmid transfected and virus infected cells. Therefore, the subcellular localization of UL6 was investigated in HSV-1 infected cells. For this sake, Vero cells were infected with HSV-1 and then IFA was carried out. As a result, UL6 also displayed dominantly nuclear localization when cells were infected at an MOI of 1 at 8 h post-infection (Figure 1D and Table 1).

Accordingly, the above data showed that UL6 localized in the nucleus regardless in live cells or chemical fixed cells, as well as in plasmid transfected cells or HSV-1 infected cells. UL6 is shown to exert certain roles that are generally associated with the nucleus, such as constituting the portal for the access of DNA into the HSV capsid, installing of a minor constituent of virions and capsids, and cleavage, disposal and encasement of duplicated viral DNA [1–9, 25]. Thus, it is no wonder that UL6 presents primarily nuclear localization.

### Identification of the nuclear localization signal of UL6

**Figure 2:**
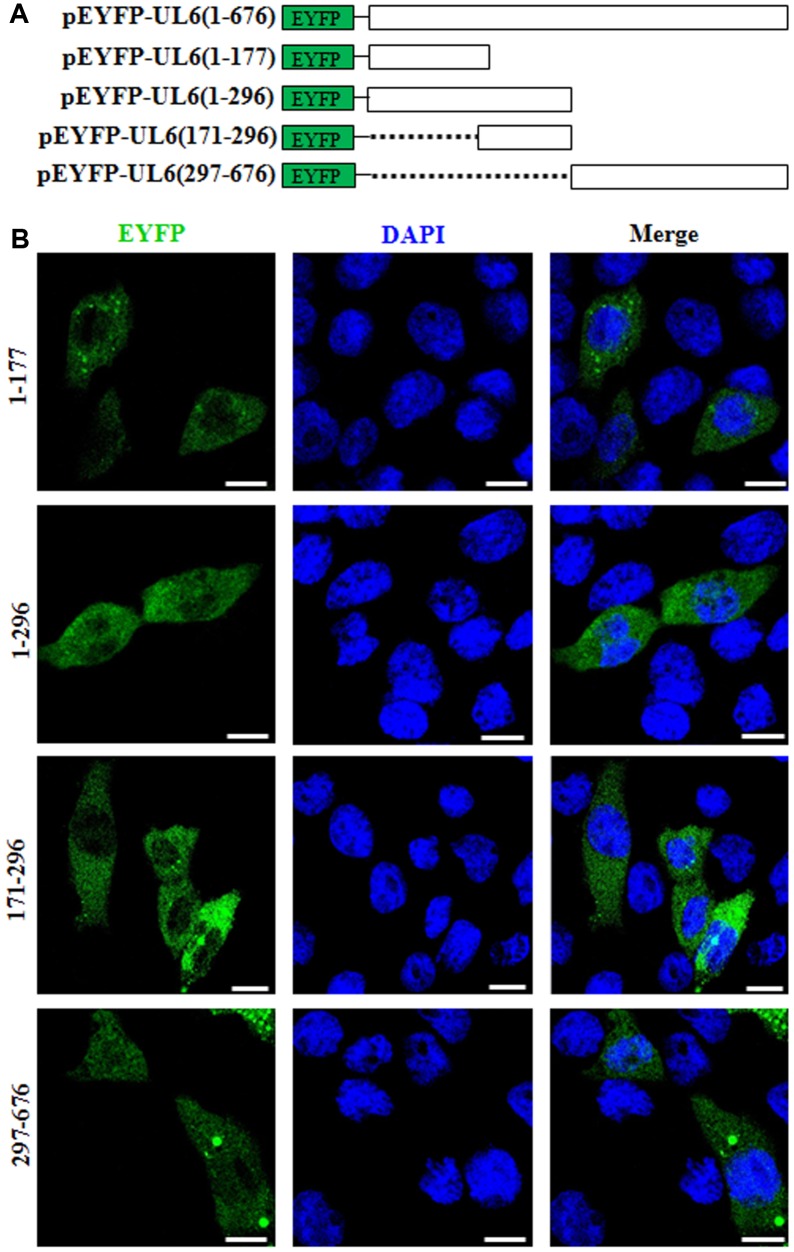
**Subcellular distribution of the UL6 deletion mutants.** (**A**) Schematic representation of wild-type UL6 protein and its N- and C-terminus deletion mutants fused with the C-terminus of EYFP. (**B**) Subcellular distribution of these UL6 deletion mutants shown in (**A**). Cells were stained with DAPI to visualize the nuclei. All scale bars indicate 10 um. Statistical analysis of the fluorescence was shown in Table 2.

Nuclear localization signal (NLS), predominantly possessed of basic residues, is vital for the nuclear accumulation of specific protein [26]. Bioinformatics analysis using PSORT II predicted that UL6 contains a potential NLS in the basic residue rich region, namely PILRKRQ at aa171-177 (pat7). However, the potential nuclear export signal of UL6 was not predicted. In order to identify the functional NLS, UL6 was firstly divided into two segments (amino acids (aa) 1-296 and aa297-676) and fused to the C-terminus of EYFP to construct aa1-296-EYFP and aa297-676-EYFP (Figure 2A). Then, these two plasmids were analyzed in COS-7 cells. As shown in Figure 2B and Table 2, the fluorescence of aa1-296-EYFP showed cytoplasmic localization, whereas aa297-676-EYFP showed pan-cellular distribution, suggesting these two regions may not contain functional NLS. To further explore the functional NLS, plasmids encoding EYFP fused to two diverse segments aa1–177 and aa171–296, which encompass the predicted NLS aa171-177, were constructed (Figure 2A) and assessed in COS-7 cells. As shown in Figure 2B and Table 2, both of the fluorescence of aa1–177-EYFP and aa171–296-EYFP were similar to that of aa1-296-EYFP, indicating the predicted NLS was non-functional, and the functional NLS of UL6 may be generated by spatial conformation.

**Table 2:** Subcellular localization of HSV-1 UL6 deletion mutants.

**Transfection of UL6 deletion mutant fused with EYFP**	**Total number of cells transfected with plasmid**	**Subcellular localization pattern of transfected plasmid**	**Number of cells with similar subcellular localization pattern**	**Percentage of cells with similar subcellular localization pattern**
1-296	30	Pan-cytoplasmic	28	93.33
1–177	30	Pan-cytoplasmic	27	90
171–296	30	Pan-cytoplasmic	28	93.33
297-676	30	Pan-cellular	26	86.67

### Characterization of the nuclear import mechanism of UL6

**Figure 3:**
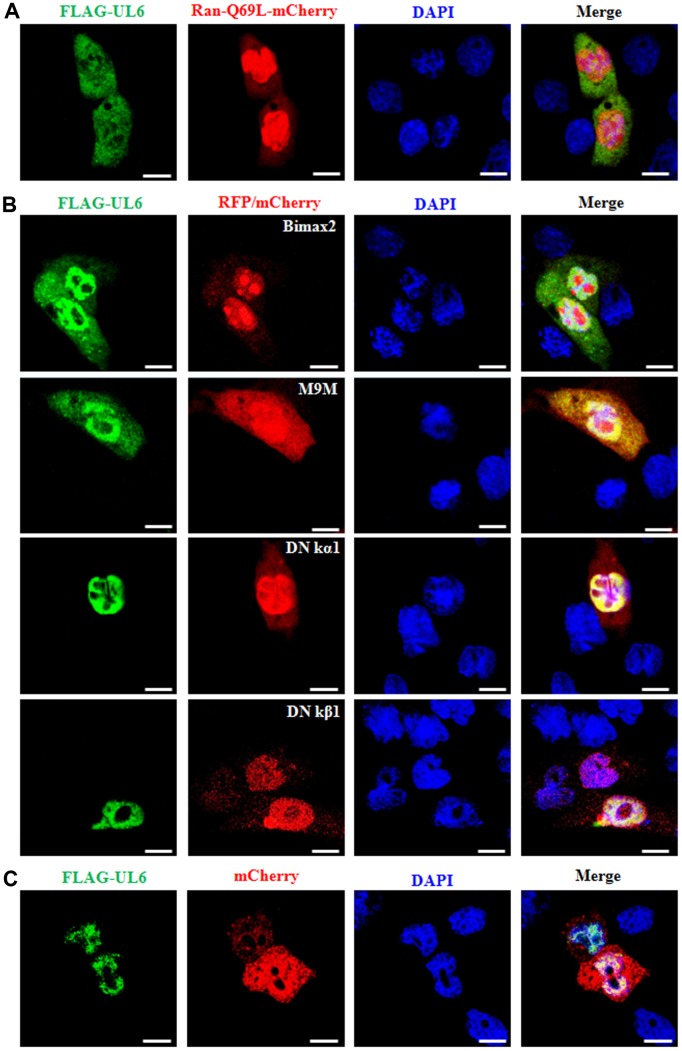
**Nuclear import mechanism of UL6.** (**A**) Fluorescence microscopy of COS-7 cells co-transfected with plasmids pFLAG-UL6 and pRan-Q69L-mCherry. (**B**) Fluorescence microscopy of COS-7 cells co-transfected with plasmid pFLAG-UL6 and plasmid encoding Bimax2-RFP, M9M-RFP, DN kα1-mCherry or DN kβ1-mCherry. (**C**) Fluorescence microscopy of COS-7 cells co-transfected with pFLAG-UL6 and pmCherry-N1. FITC-labeled proteins and mCherry fusion proteins were shown in its original color green and red, respectively, and the merged image was presented in yellow signal. All scale bars indicate 10 um, Statistical analysis of the fluorescence was shown in Table 3.

To date, Ran GTPase is reported to be indispensable for the nuclear transport process of most nuclear target protein [27]. To probe the nuclear import mechanism of UL6, the dominant negative (DN) mutant of RanGTP, with deficiency in GTP hydrolysis (Ran-Q69L) [28], was utilized to inspect whether Ran participates in the nuclear translocation of UL6. Plasmids expressing Ran-Q69L-mCherry and FLAG-UL6 were co-transfected into COS-7 cells, then their subcellular distributions were analysed by IFA. As a result, co-transfection of Ran-Q69L significantly abolished the nuclear accumulation of UL6 (Figure 3A and Table 3). Considering the evolutionary conserved nuclear pore complex (NPC) only endorses the dispersion of small proteins with approximate molecular masses of 40~60 kDa [29, 30], and FLAG-UL6 has a molecular mass of about 76 kDa, it cannot be proposed to export the nucleus by simple dispersion. Consequently, UL6 is a Ran-associated protein and is transported into the nucleus from the cytoplasm through a canonical nuclear transport pathway mediated by GTP hydrolysis.

**Table 3:** Nuclear import mechanism of HSV-1 UL6.

**Transfected viral gene**	**Co-transfected vector, DN mutant or inhibitor of nuclear import related receptor**	**Total number of cells co-transfected with two plasmids (viral gene and vector, DN mutant or inhibitor of nuclear import related receptor)**	**Subcellular localization change of viral gene in cells co-transfected with two plasmids**	**Subcellular localization pattern of viral gene in cells co-transfected with two plasmids**	**Number of subcellular localization change of viral gene in cells co-transfected with two plasmids**	**Percentage of subcellular localization change of viral gene in cells co-transfected with two plasmids**
FLAG-UL6	DN Ran-mCherry	30	Yes	Pan-cellular	28	93.33
FLAG -UL6	RFP-Bimax2	30	Yes	Pan-cellular, with slightly more fluorescence in nucleus	23	76.67
FLAG -UL6	RFP-M9M	30	Yes	Pan-cellular, with slightly more fluorescence in nucleus	25	83.33
FLAG -UL6	DN kα1-mCherry	30	No	Predominantly nuclear	2	6.67
FLAG -UL6	DN kβ1-mCherry	30	No	Predominantly nuclear	1	3.33
FLAG -UL6	mCherry	30	No	Predominantly nuclear	0	0

About the nuclear translocation, the NLS of cargo is bound by different members of the importin family. In heterodimer importin α/β, importin α binds the NLS of a specific cargo, while importin β is responsible for the conformational alteration of importin α, to reinforce the interaction of importin α-NLS [31]. Then, the importin α/β-cargo complex traffics into the nucleus and is detached by the combination of importin β1 (karyopherin β1, kβ1) with Ran-GTP [32]. In mammals, the subcellular transport involves at least six cellular transporters [33, 34], namely importin α1 (karyopherin α2, kα2), importin α3 (karyopherin α4, kα4), importin α4 (karyopherin α3, kα3), importin α5 (karyopherin α1, kα1), importin α6 (karyopherin α5, kα5) and importin α7 (karyopherin α6, kα6). Besides, the NLS-containing cargo also can be directly bound by diverse importin β members [35, 36]. To identify the cellular transporter for the nuclear targeting of UL6, the expression plasmids of importin α1, α3, α6 and α7 nuclear transport inhibitor Bimax2 [37], transportin-1 (importin β2) nuclear import inhibitor M9M [38], importin β association deficient mutant of importin α5 (DN kα1) [39] and Ran binding deficient mutant of importin β1 (DN kβ1) were co-transfected with FLAG-UL6 expression plasmid, respectively. As results (Figure 3B and Table 3), COS-7 cells were transfected with plasmid expressing EYFP-UL6 or EYFP for 24 h, then cells were examined by confocal fluorescence microscopy. In addition, COS-7 cells were transfected with plasmid expressing Flag-UL6 for 24 h, and Vero cells were infected with HSV-1 (F strain) at an MOI of 1 for 8 h, then cells were subjected to IFA analysis using anti-Flag mAb or anti-UL6 pAb.

COS-7 cells were transfected with plasmid expressing UL6 deletion mutants 1-296, 297-676, 1–177 and 171–296 fused to the C-terminus of EYFP. 24 h post-transfection, cells were examined by confocal fluorescence microscopy.

Expression plasmid of FLAG-UL6 was co-transfected with the plasmid expressing Ran-Q69L-mCherry, RFP-Bimax2, RFP-M9M, DN kα1-mCherry, DN kβ1-mCherry or mCherry into COS-7 cells. 24 h post-transfection, cells were examined for the subcellular localization of UL6 by confocal fluorescence microscopy.

co-transfection of Bimax2 and M9M could efficiently diminished the nuclear import of UL6, whereas DN kα1 or DN kβ1 did not obviously lessened the nuclear trafficking of UL6. As negative control, UL6 was not relocalized by mCherry when COS-7 cells were co-transfected with pCMV-Flag-UL6 and mCherry vector (Figure 3C and Table 3). These data revealed that the nuclear transport of UL6 was mediated by transportin-1, and may be one of the cellular transporters of importin α1, α3, α6 and α7, but not importin α5 or importin β1.

### UL6 interacts with transportin-1, importin α1 and importin α7

**Figure 4:**
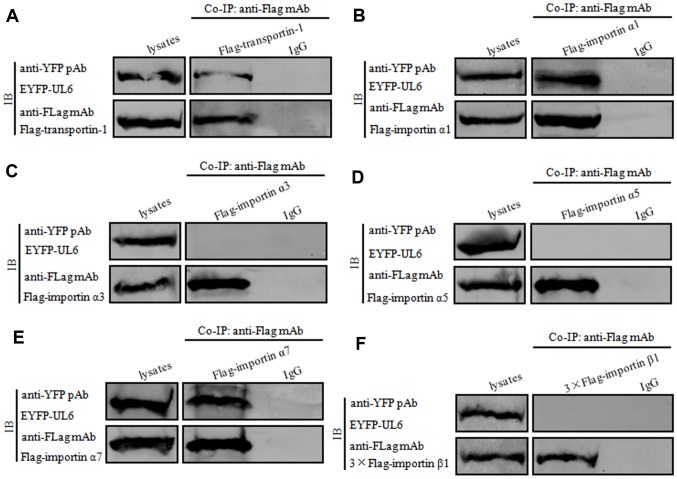
**UL6 binds transportin-1, importin α1 and importin α7.** (**A**–**F**) Co-IP of UL6 with Transportin-1 (**A**), importin α1 (**B**), importin α3 (**C**), importin α5 (**D**), importin α7 (**E**) or importin β1 (**F**). pEYFP-UL6 was co-transfected with plasmid expressing pFLAG-CMV-transportin-1 (**A**), Flag-kα2 (importin α1) (**B**), Flag-kα4 (importin α3) (**C**), Flag-kα1 (importin α5) (**D**), Flag-kα6 (importin α7) (**E**) or pCMV9-3×Flag-importin β1 (**F**) into HEK293T cells. 24 h post-transfection, cells were lysed and Co-IPed with anti-Flag mAb or mouse IgG control. Cell lysates and the Co-IPed proteins were separated in denaturing 10% SDS-PAGE, and analyzed by IB with anti-Flag mAb or anti-YFP pAb.

To further verify the assumption mentioned above, plasmids combination of pFLAG-CMV-transportin-1/pEYFP-UL6, Flag-kα2 (importin α1)/pEYFP-UL6, Flag-kα4 (importin α3)/pEYFP-UL6, Flag-kα1/pEYFP-UL6, Flag-kα6 (importin α7)/pEYFP-UL6 or pCMV9-3×Flag-importin β1/pEYFP-UL6 were co-transfected into HEK293T cells for 24 h, then cell lysates were collected and co-immunoprecipitation (Co-IP) was implemented using anti-Flag mAb or mouse IgG. As results, UL6 was efficiently Co-IPed with transportin-1 (Figure 4A), importin α1 (Figure 4B) and importin α7 (Figure 4E), but not, importin α3 (Figure 4C), importin α5 (Figure 4D) or importin β1 (Figure 4F). In contrast, no target protein was Co-IPed by IgG (Figure 4), illustrating UL6 could interact with transportin-1, importin α1 and importin α7.

### Verification of the nuclear import mechanism of UL6

**Figure 5:**
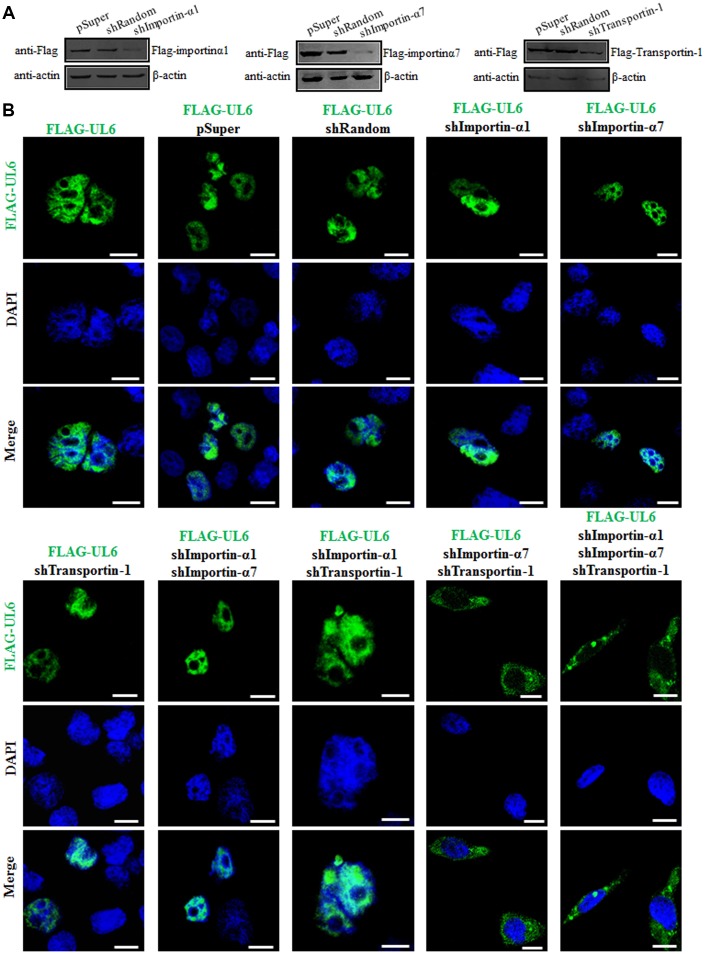
**Subcellular distribution of UL6 in presence of different shRNA expression plasmids.** (**A**) Verification of knock down efficiency of the constructed shRNA expression plasmids for importin α1, importin α7 and transportin-1. HEK293T cells were co-transfected with the plasmids combination of Flag-kα2 (importin α1)/pSuper, Flag-kα2/shRandom, Flag-kα2/shImportin-α1, Flag-kα6 (importin α7)/pSuper, Flag-kα6/shRandom, Flag-kα6/shImportin-α7, pFLAG-CMV-transportin-1/pSuper, pFLAG-CMV-transportin-1/shRandom or pFLAG-CMV-transportin-1/shTransportin-1 for 24 h. Then, cells were lysed and IB was performed with anti-Flag mAb. β-actin was used as a loading control. (**B**) One or two or three plasmids of shImportin-α1, shImportin-α7 and shTransportin-1 were co-transfected with pFLAG-UL6 into COS-7 cells for 24 h, then IFA was carried out using confocal fluorescence microscopy. Statistical analysis of the fluorescence was shown in Table 4.

To finally validate the nuclear import mechanism of UL6, short hairpin RNA (shRNA) expression plasmids were constructed to knock down the expression of importin α1, importin α7 and transportin-1. Compared to the shRNA control vector (shRandom), shImportin-α1, shImportin-α7 and shTransportin-1 could effectively knock down the expression of importin α1, importin α7 and transportin-1, respectively (Figure 5A), suggesting the related shRNA expression plasmids were successfully constructed. Then, one or two or three plasmids combination of shImportin-α1, shImportin-α7 and shTransportin-1 were co-transfected with pFLAG-UL6 into COS-7 cells and IFA was carried out to analyze whether these shRNA expression plasmids can influence the nuclear import of UL6. As results, the nuclear translocation of UL6 was not obviously affected when one of importin α1, importin α7 and transportin-1, or two of importin α1/importin α7 and importin α1/transportin-1, were knocked down. However, the nuclear trafficking of UL6 was significantly inhibited when importin α7/transportin-1 or importin α1/importin α7/transportin-1 were simultaneously knocked down (Figure 5 and Table 4), confirming UL6 could be imported into the nucleus via various transport pathways, which was primarily mediated by importin α7 and transportin-1*.*

**Table 4:** Verification of the nuclear import mechanism of HSV-1 UL6.

**Transfected viral gene**	**Co-transfected with one or two or three plasmids of shImportin α1, shImportin α7 and shTransportin-1**	**Total number of cells with FLAG-UL6 fluorescence**	**Subcellular localization change of cells with FLAG-UL6 fluorescence**	**Subcellular localization pattern of cells with FLAG-UL6 fluorescence**	**Number of subcellular localization change of cells with FLAG-UL6 fluorescence**	**Percentage of subcellular localization change of cells with FLAG-UL6 fluorescence**
FLAG-UL6	shVector	30	No	Predominantly nuclear	0	0
FLAG-UL6	shRandom	30	No	Predominantly nuclear	0	0
FLAG-UL6	shImportin-α1	30	No	Predominantly nuclear	1	3.33
FLAG-UL6	shImportin-α7	30	No	Predominantly nuclear	1	3.33
FLAG-UL6	shTransportin-1	30	No	Predominantly nuclear	2	6.67
FLAG-UL6	shImportin-α1+ shImportin-α7	30	No	Predominantly nuclear	3	10
FLAG-UL6	shImportin-α1+ shTransportin-1	30	No	Predominantly nuclear	3	10
FLAG-UL6	shImportin-α7+ shTransportin-1	30	Yes	Pan-cytoplasmic	26	86.67
FLAG-UL6	shImportin-α1+ shImportin-α7+shTransportin-1	30	Yes	Pan-cytoplasmic	28	93.33

shVector, shRandom, one or two or three plasmids of shImportin-α1, shImportin-α7 and shTransportin-1 were co-transfected with FLAG-UL6 into COS-7 cells. 24 h post-transfection, cells were examined for the subcellular localization of UL6 by IFA using confocal fluorescence microscopy.

As we known, HSV-1 encodes more than 80 structural proteins, some of which need to be transported into the nucleus for their functions execution, such as promoting viral proliferation, restraining host transcription and expression, inhibiting host innate immunity, etc. The nuclear accumulation of these proteins is mediated by one or more different nuclear import receptors, of course including importin α1, importin α7 and transportin-1. In addition, some host proteins also need to be transported into the nucleus by different nuclear import receptors, to perform their corresponding functions. Therefore, it is bound to affect the nuclear accumulation of many proteins of HSV-1 (and host) when the DN mutants of importin α 1, importin α 7 and transportin-1 are transfected into cells or these nuclear import receptors are knocked down by shRNA expression plasmid. Consequently, it is difficult for us to determine whether the reduction of DNA replication, nucleocapsid assembly and virions production of HSV-1 is the direct outcome of the inhibition of UL6 nuclear translocation.

In conclusion, we had proved that UL6 was a genuine nuclear localization protein. Although the predicted NLS of UL6 was nonfunctional, it was identified to be transported into the nucleus through Ran-, transportin-1-, importin α1- and importin α7-dependent nuclear import mechanism, which was largely mediated by the later two nuclear import receptor. These results dissected the molecular determinant for the nuclear transport of UL6, and will shine light for the further study of its biological roles during HSV-1 infection.

## MATERIALS AND METHODS

### Plasmids construction

DNA polymerase KOD-Plus-Neo, restriction enzyme and T4 DNA ligase that involved in molecular cloning were supplied by TOYOBO (Osaka, Japan), New England Biolabs (MA, USA) and Takara (Dalian, China), respectively. The UL6 ORF of HSV-1 (F strain) was amplified from plasmid template pYEbac102 [40] and inserted into pEYFP-C1 (Clontech) to yield pEYFP-UL6, as described in our previous studies [13, 15, 18, 22–24, 41]. Subsequently, the UL6 ORF of pEYFP-UL6 was subcloned into pFLAG-CMV-2 (Sigma) to produce pCMV-Flag-UL6. The deletion mutants of UL6 fused to the C-terminus of EYFP were constructed with similar method, and the related primers used for UL6 are available upon request. In addition, the shRNAs for importin α1 (5′-CTACCTCTGAAGGCTACACTT-3′), importin α7 (5′-CCTGTGTTGATCGAAATCCTT-3′), transportin-1 (5′-CCGTACTGTGAACCTGTGTAT-3′) and a control shRNA (shRandom, 5′-CTCAACTCACGTGTCTAGTGTC-3′) were inserted into pSUPER.retro.puro (shVector) (BD Biosciences) to construct pSUPER-shImportin α1 (shImportin-α1), pSUPER-shImportin α7 (shImportin-α7), pSUPER-shTransportin-1 (shTransportin-1) and pSUPER-shRandom (shRandom), respectively.

pRan-Q69L-mCherry, pFLAG-CMV-transportin-1, pDN kα1-mCherry and pDN kβ1-mCherry were described in our previous studies [14–19, 21, 22, 24]. Plasmids expressing RFP-M9M and RFP-Bimax2 were generously offered by Dr. Nobuyuki Nukina [42], and other plasmids were afforded as indicated by Dr. Yoshihiro Yoneda [43] (Flag-kα1 and Flag-kα6), Dr. Riku Fagerlund [44] (Flag-kα2 and Flag-kα4) and Dr. Ben Margolis (pCMV9-3×Flag-importinβ1).

### Plasmid transfection and fluorescence analysis

Plasmid transfection and fluorescence analysis were carried out, as described in our previous studies [13, 15, 18, 22–24, 41, 45]. Briefly, COS-7 cells were transiently transfected with the indicated plasmid DNA mixed with Thermo Scientific TurboFect Transfection Reagent in line with the manufacturer’s instructions. 24 h post-transfection, DAPI staining, which is widely applied in our previous studies of related fluorescent experiments [12–15, 17, 18, 20, 21], was employed to investigate whether the target protein locates in the nucleus or in the cytoplasm. Then, cells were analyze by live cells fluorescence microscopy or IFA, using a laser scanning confocal microscopy (Leica SP8). The image shown represents a great proportion of the cells with homogeneous subcellular distribution. EYFP fusion proteins were shown in pseudocolor green, FITC-labeled proteins and mCherry fusion proteins were shown in their original colors green and red, respectively, and the merged image was presented in yellow signal. All scale bars indicate 10 um, and images were processed using Adobe Photoshop.

### Virus infection and IFA

Vero cells infected with HSV-1 (MOI=1) for 8 h were fixed with 4% paraformaldehyde, permeabilized with 0.5% Triton X-100, and stained with the anti-UL6 polyclonal antibody (pAb) [46]. Then, cells were incubated with fluorescein isothiocyanate (FITC)-conjugated goat anti-rabbit immunoglobulin G (Zymed Laboratories) and stained with DAPI. Cells were finally detected with a laser scanning confocal microscopy. All scale bars indicate 10 um, and images were processed with Adobe Photoshop.

### Co-IP and immunoblotting

Co-IP and immunoblotting (IB) assays were manipulated as described previously [13, 15, 18, 22–24, 41, 47, 48]. Summarily, HEK293T cells were co-transfected with FLAG- or EYFP-tagged expression plasmids for 24 h. Cells were then collected and lysed on ice with 1 mL of lysis buffer. The lysate was subsequently incubated with anti-Flag monoclonal antibody (mAb, Sigma) or nonspecific mouse control antibody (IgG) and a 1:1 slurry of Protein A/G PLUS-Agarose (Santa Cruz Biotechnology) for at least 4 h or overnight at 4 ^o^C. Then, lysis buffer was used to wash beads for three times. Finally, cell lysates and the Co-IPed proteins, were subjected to IB analysis with anti-Flag mAb and anti-YFP pAb (Santa Cruz Biotechnology). All Co-IP were duplicated at least two times, and analogous data were obtained.
